# Supplementary material for: Combining transfer learning with retinal lesion features for accurate detection of diabetic retinopathy
Source: Front Med (Lausanne). 2022 Nov 8;9:1050436. doi: 10.3389/fmed.2022.1050436 (PMC9681494; doi:10.3389/fmed.2022.1050436)
Supplement: Supplementary file 2 [file Data_Sheet_2.docx]

Supp_Figure 1

Supp_Figure 2


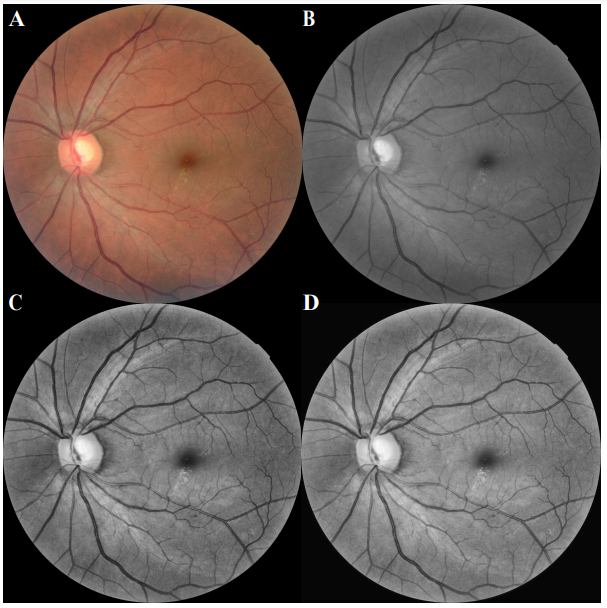


**Supp_Figure 3**

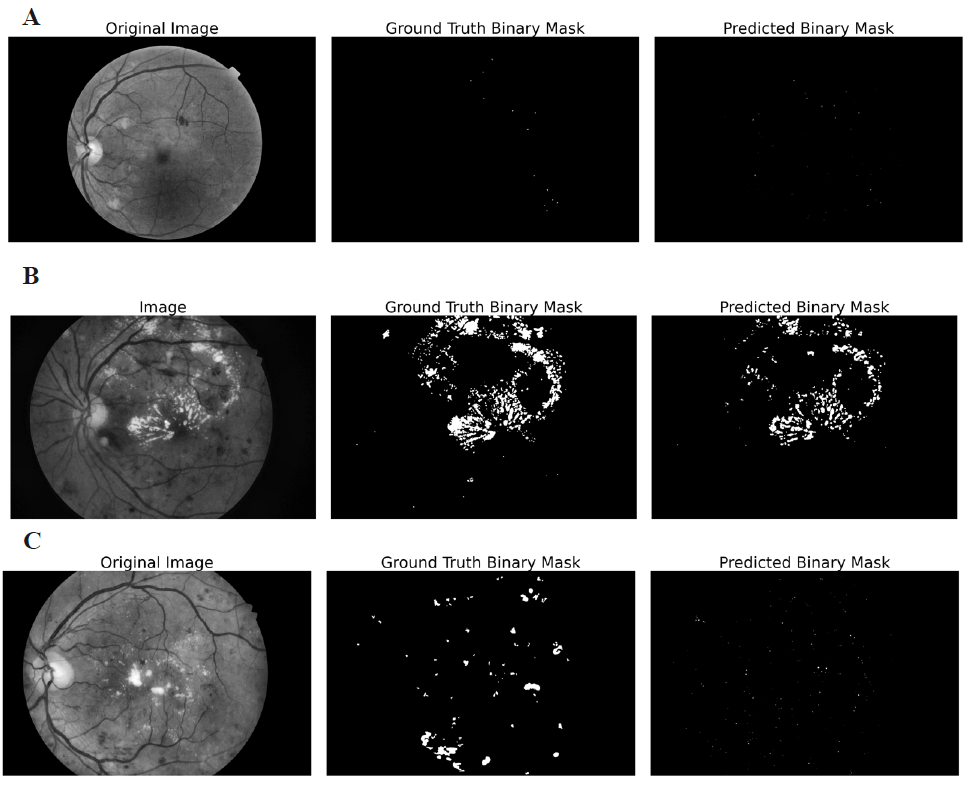

A= An example UNET model predictions of Microaneurysms

B= UNET model predictions of Exudates

C= UNET model predictions of Hemorrhages
